# Retraction: Efficient removal of a typical dye and Cr(vi) reduction using N-doped magnetic porous carbon

**DOI:** 10.1039/d6ra90060g

**Published:** 2026-07-06

**Authors:** Shouwei Zhang, Xiangxue Wang, Jiaxing Li, Tao Wen, Jinzhang Xu, Xiangke Wang

**Affiliations:** a School of Materials Science and Engineering, Hefei University of Technology Hefei P. R. China; b Key Laboratory of Novel Thin Film Solar Cells, Institute of Plasma Physics, Chinese Academy of Sciences P.O. Box 1126 Hefei P. R. China lijx@ipp.ac.cn xkwang@ipp.ac.cn +86-551-65591310 +86-551-65593308; c Faculty of Engineering, King Abdulaziz University Jeddah Saudi Arabia

## Abstract

Retraction of ‘Efficient removal of a typical dye and Cr(vi) reduction using N-doped magnetic porous carbon’ by Shouwei Zhang *et al.*, *RSC Adv.*, 2014, **4**, 63110–63117, https://doi.org/10.1039/C4RA10189H.

We, the authors, hereby wholly retract this *RSC Advances* article due to concerns with the reliability of the XRD patterns in Fig. 1a.

In Fig. 1a, the XRD patterns for N-MPC and Ni@GM + KOH are partially identical. We repeated the experiments and provided new correct data, and we believe that this does not affect the discussion and conclusions of the original article. However, it is not possible to replace the published data with the new data. Therefore, to protect the rigor of the scientific record, we have decided to retract this article and apologize for any inconvenience to readers.

Shouwei Zhang has stated that all authors agree to this retraction.

Signed: Jiaxing Li, Shouwei Zhang, Tao Wen, Xiangke Wang

Date: 18th May 2026

Retraction endorsed by Laura Fisher, Executive Editor, *RSC Advances*.

